# Comparative Metabolomic Analysis and Antinociceptive Effect of Methanolic Extracts from *Salvia cinnabarina*, *Salvia lavanduloides* and *Salvia longispicata*

**DOI:** 10.3390/molecules29225465

**Published:** 2024-11-20

**Authors:** Nancy Ortiz-Mendoza, Juan L. Monribot-Villanueva, José A. Guerrero-Analco, Martha J. Martínez-Gordillo, Francisco A. Basurto-Peña, Eva Aguirre-Hernandez, Marcos Soto-Hernández

**Affiliations:** 1 Laboratorio de Productos Naturales, Departamento de Ecología y Recursos Naturales, Facultad de Ciencias, Universidad Nacional Autónoma de México, Mexico City 04510, Mexico; nancy_om@ciencias.unam.mx; 2 Posgrado en Ciencias Biológicas, Unidad de Posgrado, Edificio D, 1° Piso, Circuito de Posgrados, Ciudad Universitaria Coyoacán, Mexico City 04510, Mexico; 3 Red de Estudios Moleculares Avanzados, Instituto de Ecología A. C., Carretera Antigua a Coatepec 351, Xalapa 91073, Mexico; juan.monribot@inecol.mx; 4 Departamento de Biología Comparada, Herbario de la Facultad de Ciencias, Universidad Nacional Autónoma de México, Mexico City 04510, Mexico; mjmg@ciencias.unam.mx; 5 Jardín Botánico, Instituto de Biología, Universidad Nacional Autónoma de México, Mexico City 04510, Mexico; abasurto@ib.unam.mx; 6 Posgrado en Botánica, Colegio de Postgraduados, Campus Montecillo, Texcoco 56264, Mexico; msoto@colpos.mx

**Keywords:** antinociceptive effect, *Salvia*, terpenoids, flavonoids, UPLC-MS

## Abstract

Mexico is considered one of the countries with the greatest diversity of the *Salvia* genus. A significant percentage of its species are known for their use in traditional medicine, highlighting their use as an analgesic. The objective of this work was to determine the chemical composition of the methanolic extracts of *S. cinnabarina*, *S. lavanduloides* and *S. longispicata* through untargeted metabolomics, as well as the in vivo evaluation of the antinociceptive effect and acute oral toxicity. The chemical profiling was performed using ultra-high performance liquid chromatography coupled with a high-resolution mass spectrometry (UPLC-ESI^+/−^-MS-QTOF) system and tentative identifications were performed using a compendium of information on compounds previously isolated from Mexican species of the genus. Pharmacological evaluation was carried out using the formalin test and OECD guidelines. The analysis of the spectrometric features of the mass/charge ratios of the three salvias shows that a low percentage of similarity is shared between them. Likewise, the putative identification allowed the annotation of 46 compounds, mainly of diterpene and phenolic nature, with only four compounds shared between the three species. Additionally, the extracts of the three salvias produced a significant antinociceptive effect at a dose of 300 mg/kg administered orally and did not present an acute oral toxicity effect at the maximum dose tested, indicating a parameter of LD_50_ > 2000 mg/kg. The exploration of the chemical profile of the three salvias by untargeted metabolomics shows that, despite being species with antinociceptive potential, they have different chemical profiles and therefore different active metabolites.

## 1. Introduction

The species of the genus *Salvia* L. have been used throughout the world for their broad spectrum of biological activities, to name a few, they have been used for the treatment of digestive problems, cardiovascular and cerebrovascular diseases, pain, bronchitis, cough, asthma, inflammation, depression, anxiety, insomnia, and skin conditions [1,2,3,4]. Pharmacological studies report its antioxidant, antidiabetic, antiviral, antinociceptive, anti-inflammatory, anti-Alzheimer, and antitumor properties, among others. Terpenoids, phenolic acids, and flavonoids are compounds responsible for the medicinal effects of sage [2,3,4,5,6,7,8]. Regarding the analgesic properties of *Salvia*, there is evidence in the literature of its effect through different models of nociceptive and inflammatory pain in rodents. The ethanolic extract of *S. plebeia* R.Br. presents pharmacological activity in the abdominal pain model and in the inflammatory process induced in the carrageenan test [9]. The hydroalcoholic extract of *S. officinalis* L. produces antinociceptive and anti-inflammatory effects in abdominal stretching, formalin and carrageenan tests [10]. Likewise, the hydroalcoholic extract of *S. miltiorrhiza* Bunge has analgesic and anti-inflammatory effects using the collagen-induced arthritis model [7]. Similarly, the ethanolic extract of *S. lachnostachys* Benth. had good activity both in the formalin test and the anti-arthritis model [11].

Mexico is considered one of the areas with the greatest diversity of the *Salvia* genus in the world, represented by around 307 species [12]. Almost all Mexican salvias (sages) species are included within the subgenus *Calosphace* [13,14]. From ancient times to the present, various species of *Salvia* have been known for their wide range of ornamental, cosmetic, culinary and medicinal uses [15,16,17,18]. The last one is the most notable, as there are around 56 species belonging to the subgenus *Calosphace* used in traditional Mexican medicine to treat various diseases of the digestive system, nervous disorders, pregnancy, childbirth and postpartum, and other culture-bound syndromes [19]. Within the wide spectrum of properties of traditionally used Mexican salvias, analgesic stands out [16,19,20]. Some examples of sages with this characteristic are *S. microphylla* Kunth, *S. coccinea* Buc’hoz ex Etl., *S. lavanduloides* Kunth, *S. elegans* Vahl, *S. polystachia* Cav., *S. leucantha* Cav., *S. mexicana* L., *S. hispanica* L., *S. amarissima* Ortega, and *S. tiliifolia* Vahl, of which the aerial part is used, prepared as an infusion, to treat pain, mainly in south-central Mexico (Yucatan, Chiapas, Oaxaca, Guerrero, Morelos, Michoacan) [21,22,23,24,25,26,27,28,29,30]. Despite the analgesic properties of Mexican salvias, most in vitro, in vivo and ex vivo studies, both of extracts and isolated compounds, have been mainly directed to the evaluation of antimicrobial and cytotoxic effects [31,32,33]. Only the species *S. amarissima* (Syn. *S. circinate* Cav.), *S. purpurea* Cav., *S. semiatrata* Zucc., and *S. tiliifolia* of the subgenus *Calosphace* have been evaluated in antinociception models. Studies have reported no acute oral toxicity of the extracts and a significant reduction in nociception of extracts of different polarity, at doses between 100 and 300 mg/kg, administered orally (p.o.), as well as of isolated compounds of diterpene nature (amarisolide A, tilifodiolide and 7-keto-neoclerodan-3,13-dien-18,19:15,16-diolide) and phenolic (pedalitin) at doses of 1–10 mg/kg, p.o. [34,35,36,37]. The potent antinociceptive effect of compounds of this nature, isolated from other botanical families, has been corroborated in in vivo tests [37,38,39,40]. In relation to the above, it should be noted that various phytochemical studies of approximately 50 species of *Salvia* of the subgenus *Calosphace* have reported the presence of secondary metabolites of terpene and phenolic nature, with diterpenes (abietane, clerodane, labdane and pimarane) being the most abundant, even postulated as chemotaxonomic markers [1,19,41,42]. *S. cinnabarina* M.Martens & Galeotii, *S. lavanduloides* and *S. longispicata* M.Martens & Galeotii, are three species of the subgenus *Calosphace*, used in tea form for analgesic purposes in traditional Mexican medicine and that have a wide distribution in Mexican territory, with the exception of the Baja California peninsula [43]. There are no reports on the antinociceptive potential in in vivo models and little is known about its phytochemistry. Therefore, in this study, the chemical composition is explored by means of an untargeted metabolomic analysis, using ultra-high performance liquid chromatography coupled with high-resolution mass spectrometry (UPLC-ESI^+/−^-MS-QTOF), for the identification of a significant number of specialised metabolites in the selected species. Likewise, acute oral toxicity is evaluated following the Organisation for Economic Co-operation and Development (OECD) Guidelines for the Testing of Chemicals (2001), and antinociceptive activity using the formalin test of methanol extract by oral route [44].

## 2. Results

### 2.1. Chemical Profiling via Ultra Performance Liquid Chromatography Coupled to Mass Spectrometry Quadrupole Time of Flight (UPLC-ESI^+/−^-MS-QTOF)

#### 2.1.1. Preliminary Comparison of the Retention Time-Mass/Charge Features Between *Salvia* Species

**Figure 1:**
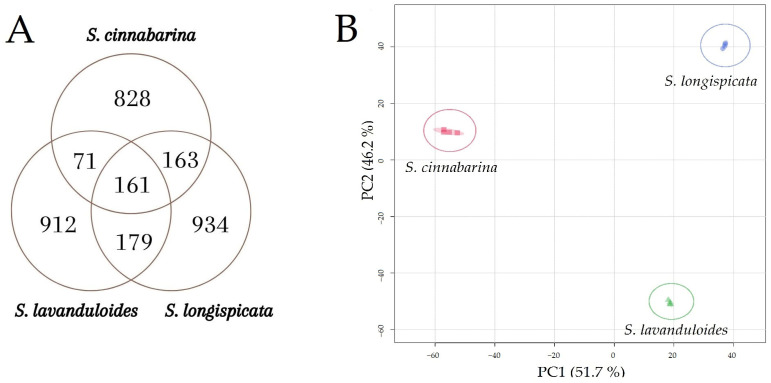
Comparative analysis by Venn diagram (**A**) and PCA (**B**) of the constitution of the rt-*m*/*z* features of the methanolic extracts of *S. cinnabarina*, *S. lavanduloides* and *S. longispicata* obtained by UPLC-ESI^+/−^-MS-QTOF.

A preliminary comparative analysis of the constitution of retention time-mass/charge (rt-*m*/*z*) features obtained by UPLC-ESI^+/−^-MS-QTOF, of the three sages, shows that they share a low percentage of similarity between them. This is visualised by the Venn diagram (Figure 1A) and the principal component analysis (PCA) (Figure 1B), which indicate that the three salvias do not group together by similarity in their rt-*m*/*z* profile.

#### 2.1.2. Difference in Chemical Composition of Sages

The experimental *m*/*z* values obtained from the three sages compared with the data reported from molecules previously isolated from Mexican *Salvia* species allowed the tentative identification of 46 metabolites including five phenolic acids, 13 flavonoids, 24 diterpenes and four triterpenes. Table 1 shows the chromatographic (rt) and spectrometric (*m*/*z*) data for each compound.

It is important to note that when comparing the historical data of compounds previously isolated from Mexican salvias with the experimental data, only 46 data points were correlated with a mass error of less than 5 ppm in negative mode and one in positive mode. The positive value corresponds to kaempferol, which is also identified in negative mode. Both are indicated as compound 18 with the following notation: Kaempferol*, in positive mode, was identified in *S. cinnabarina* and Kaempferol**, in negative mode, in *S. lavanduloides*.

The most relevant results are briefly mentioned. Diterpenes were the group with the highest number of molecules identified in the extracts of sages (Table 1, No. **19**–**42**), detected in rt from 6.54 to 12.16 min. The abietanes and clerodanes stand out with 14 and 8 structures, respectively. The abietanes (**29**, **30**, **32**–**34**, **36**), clerodane **23**, pimarane **41** and labdane **42** were identified in *S. cinnabarina*. The abietanes (**27**, **28**, **34**, **35**, **38**–**40**) and the clerodanes, amarisolide F (**19**) and salvixalapadiene (**21**) were present in *S. longispicata*. Furthermore, seven clerodanes (**19**–**22**, **24**–**26**) and three abietanes **(27**, **31**, **37**) were detected in *S. lavanduloides*, identified as 16-hydroxycarnosic acid, 16-acetoxycarnosic acid and 7α-acetoxy-6,7 dihydroicetexone, respectively. Flavonoids were the second largest group with 13 compounds identified (**2**, **4**–**8**, **10**–**12**, **15**–**18**). Rutin (**5**) was present in all three salvias, whereas luteolin-7-O-glucuronide (**8**), apigenin (**12**) and 5,6-dihydroxy-7,3′,4′-trimethoxy (**17**) were only present in *S. cinnabarina*. Schaftoside (**2**), quercetin (**11**) and kaempferol (**18**) were found in *S. lavanduloides*. Also, cyanidin 3,5-diglucoside (**4**) and miquelianin (**6**) were identified only in *S. longispicata*. Phenolic acids were the third group identified (**1**, **3**, **9**, **13** and **14**) at a retention time of 0.52, 3.41, 4.67, 5.2 and 5.37 min, respectively. Compound **1**, identified as sagerinic acid, represented by the molecular ion *m*/*z* 719.1643, was detected only in *S. cinnabarina*. Syringic acid (**3**) with an ion *m*/*z* at 197.0449 was found in *S. longispicata* and salvianolic acid A (**14**) with *m*/*z* at 493.1141 in *S. lavanduloides*. Rosmarinic acid (**13**) with an ion *m*/*z* at 359.0775 was present in all three salvias. Finally, triterpenoids were identified as a fourth group, an oleanane derivative (**43**) was only present in *S. cinnabarina*, while 3-hydroxyestran-17-one (**44**) and an ursane derivative (**45**) were found in all three salvias (Table 1).

**Figure 2:**
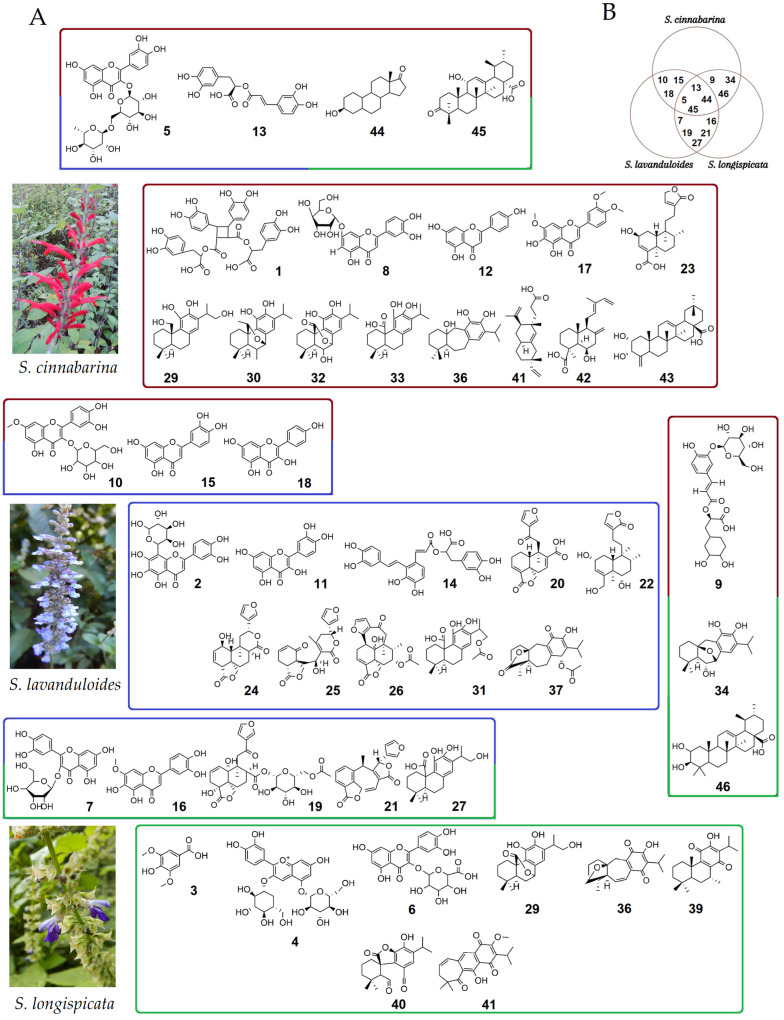
Metabolites present in the analysed *Salvia* species. The coloured blocks show the chemical structures (**A**). The Venn diagram (**B**) shows the distribution of the compounds shared between the species.

The chemical structures of the identified compounds are shown in Figure 2A. The comparative analysis of the chemical composition of the three sages using a Venn diagram (Figure 2B) showed that *S. lavanduloides* shares more compounds with *S. longispicata* (**7**, **16**, **19**, **21**, **27**), corresponding to quercetin glycoside, pedalitin, amarisolide F, salvixalapadiene, and 16-hydroxycarnosic acid, respectively. Three metabolites are common between *S. cinnabarina* and *S. longispicata*: salviaflaside (**9**), 5,6-dihydro-6α-hydroxysalviasperanol (**34**) and 2-hydroxyursolic acid (**46**). Likewise, rhamnetin 3-glucoside (**10**), luteolin (**15**) and kaempferol (**18**) were identified in *S. cinnabarina* and *S. lavanduloides*. Of these compounds, only four are shared between the three species: rutin (**5**), rosmarinic acid (**13**), 3-hydroxyestran-17-one (**44**), and 11β-hydroxy-3-oxo-urs-12-en-28-oic acid (**45**) (Figure 2B). In *S. cinnabarina*, 13 chemical constituents were identified as unique (**1**, **8**, **12**, **17**, **23**, **29**, **30**, **32**, **33**, **36**, **41**, **42**, **43**), 10 in *S. lavanduloides* (**2**, **11**, **14**, **20**, **22**, **24**, **25**, **26**, **31**, **37**), and 8 in *S. longispicata* (**3**, **4**, **6**, **28**, **35**, **38**, **39**, **40**) (Figure 2B).

**Figure 3:**
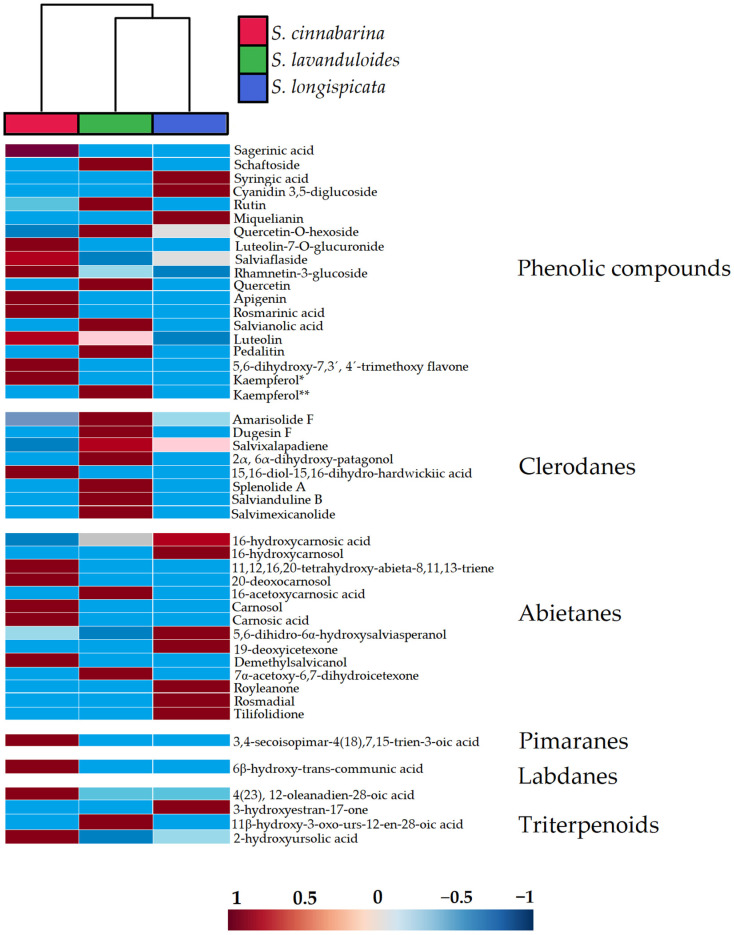
Comparative composition of *S. cinnabarina*, *S. lavanduloides*, and *S. longispicata* chemical profiles. Clustered heat map analysis of 46 metabolites. Kaempferol*: detected in positive mode. Kaempferol**: Detected in negative mode.

The differences of the 46 compounds between the three salvias are presented in a heat map (Figure 3). In this map, red indicates a relatively higher intensity of each of the compounds present in the sages and blue a lower intensity. Abietanes (**27**–**40**) and clerodanes (**19**–**26**) were the most common among salvias, with 22 compounds identified. Abietanes predominate in *S. longispicata* and *S. cinnabarina*, and clerodanes in *S. lavanduloides*. Pimarane (**41**) and labdane (**42**) were found in *S. cinnabarina*. Regarding phenolic compounds (**1**–**18**) and triterpenoids (**43**–**46**), they are present in greater quantities in *S. cinnabarina* and *S. lavanduloides*, compared to *S. longispicata* (Figure 3).

### 2.2. Acute Toxicity of the Methanol Extracts of Salvias 

**Figure 4:**
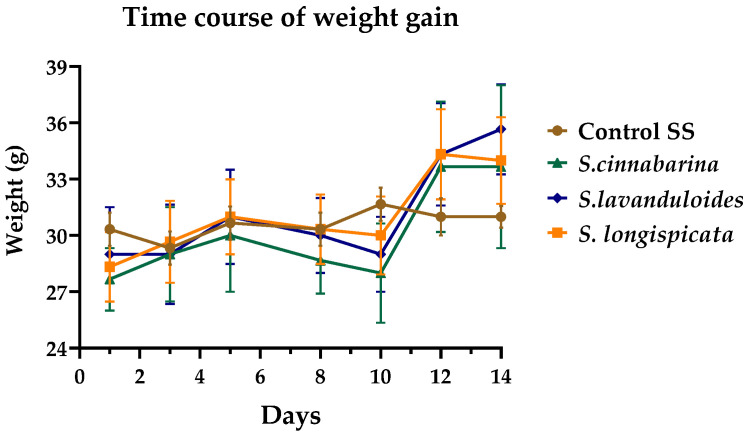
Time course of weight gain. Weight of mice recorded over 14 days in the assessment of acute oral toxicity of *S. cinnabarina*, *S. lavanduloides* and *S. longispicata* extracts. Lines represent the mean plus the Standard Error of the Mean (S.E.M.) of three animals, two-way ANOVA followed by Dunnett’s test.

The methanolic extracts of the three sages did not produce acute toxicity effects at the doses tested, nor at the maximum dose explored according to OECD’s test No. 423, indicating a parameter of LD_50_ > 2000 mg/kg, p.o. There was no significant difference in the weight of mice receiving the extract compared to the vehicle during the 14-day evaluation (Figure 4). Likewise, during the periodic observation throughout the assessment, no signs of toxicity such as changes in the skin and fur, somatomotor activity or behavioural changes were observed, nor were tremors, convulsions, diarrhoea, lethargy, sleep, or coma detected.

### 2.3. Antinociceptive Effect of Sage Extracts on the Neurogenic and Inflammatory Phases of the Formaline Test

**Figure 5:**
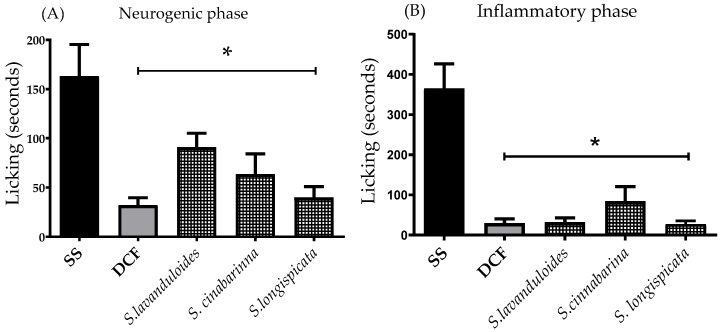
Antinociceptive-like effects of methanol extracts and the reference drug (DCF, 10 mg/kg, p.o.) in the nociceptive response of time spent in licking in the neurogenic (**A**) and inflammatory (**B**) phases after intraplantar injection of 20 µL of 1% formalin in mice. ANOVA followed by Dunnett’s post hoc test. * *p* < 0.001 indicates significant difference in comparison to the vehicle group (SS).

Methanolic extracts at a dose of 300 mg/kg, p.o. and diclofenac (DFC, reference drug at 10 mg/kg, p.o.) significantly reduced nociceptive behaviour in both the neurogenic phase (F_4,25_ = 7.571, *p* < 0.0004) (Figure 5A) and the inflammatory phase (F_4,25_ = 19.93, *p* < 0.0001) (Figure 5B) compared to the group receiving the vehicle. However, the *S. longispicata* extract showed a strong decrease in nociceptive behaviour in both phases, similar to the reference drug.

## 3. Discussion

*Salvia* is the most diverse genus within the Lamiaceae family, and Mexico is home to the largest number of species, with approximately 307 [12,70], of which around 56 species have been used in traditional Mexican medicine, with different healing properties [12,19,20]. Despite the richness of the genus and its wide use for its medicinal properties in different parts of the world [1,19,71,72], metabolomic studies are still few, mainly focused on species with some use and addressing different objectives, such as *S. miltiorrhiza* from the Old World, from the subgenus *Glutinaria*, or *S. hispanica*, from Mexico, which is within the subgenus *Calosphace* [73]. This work includes three species of the subgenus *Calosphace*, which belong to different sections and to different clades [74,75]. To explain the medicinal properties of these species, in particular their antinociceptive effect, their chemical compositions were obtained and compared with each other. Untargeted metabolomic analysis with UPLC-ESI^+/−^-MS-QTOF allowed the identification of a total of 46 compounds of phenolic and terpene nature in the extracts of salvias and also highlighted that the three species present particular chemical profiles, with only four shared compounds (rutin, rosmarinic acid, 11β-hydroxy-3-oxo-urs-12-en-28-oic acid and 3-hydroxyestran-17-one). The first three are widely distributed, both in *Salvia* [8,76,77,78] and in various botanical families [79,80,81], and this is explained because they are compounds that intervene in defence mechanisms against other organisms or that improve tolerance to certain environmental factors such as pollution, UV light and lack of water [82,83,84,85,86]. Phenolic acids such as sagerinic, syringic and salvianolic were first identified in *S. cinnabarina*, *S. longispicata*, and *S. lavanduloides*, respectively. These metabolites have been previously reported in *Salvia* species [5,8,87]. Regarding flavonoids, 13 were identified, some of which are exclusive to each sage such as schaftoside, miquelianin, and luteolin-7-O-glucoronide detected in *S. lavanduloides*, *S. longispicata* and *S. cinnabarina*, respectively. Common flavonoids of the genus were also identified, like kaemperol, pedalitin, apigenin, quercetin, luteolin, and the glycosides of the last three compounds [5,8,87]. With respect to triterpenoids, four compounds were identified in the three salvias. Previous research reports that oleanolic acid, ursolic acid, and their derivatives are common and present in almost all *Salvia* species [1,87,88,89].

Mainly terpene components are known in both *S. cinnabarina* and *S. lavanduloides*. This work contributed to expanding the phytochemical knowledge of these species by identifying the presence of structures from the group of phenolic compounds, which little is known in Mexican sages. On the other hand, it is the first time that the metabolite profile in *S. longispicata* has been investigated.

The most characteristic metabolites found in *Salvia* are the diterpenes (clerodanes and abietanes). Clerodanes are almost restricted to Neotropical salvias and are found mainly in the subgenus *Calosphace*. On the other hand, abietanes are present in European, Asian and American sages, in all subgenera, and are also found in Mexican salvias, both in the subgenus *Audibertia*, where they seem to be more abundant, and in the subgenus *Calosphace*, to which the three species studied belong. In addition, 14 abietane and eight clerodane structures were identified in this research. It should be noted that abietanes predominate in *S. longispicata* and *S. cinnabarina*, and clerodanes in *S. lavanduloides*, in the latter, two clerodanes have previously been isolated [90]. At the same time, this work reports, for the first time, the presence of five more clerodanes and two more abietanes in *S. lavanduloides*. On the other hand, five abietanes, one clerodane, one labdane, and one pimarane were identified in the extract of *S. cinnabarina*. These last two compounds have already been previously identified and isolated in this species [64,65]. It is worth noting that labdanes and pimaranes are not very abundant in *Salvia*, being identified only in *S. hispanica*, *S. parryi* A.Gray, *S. fulgens* Cav., *S. microphylla*, *S. greggii* A. Gray, *S. sclarea* L., and *S. officinalis* [1].

Specifically, the chemical profile of the three sages is contrasting, given that they are not phylogenetically close and, despite the fact that they were all collected in the State of Oaxaca, the microenvironments where they develop are different. *Salvia cinnabarina* belongs to the Incarnatae section and is the only one that synthesizes pimaranes and labdanes [64,65,91], compounds not present in the Lavanduloideae section and the Angulatae section, which respectively include *S. lavanduloides* and *S. longispicata*, so these diterpenoids could be good chemotaxonomic markers.

Flavonoids are common and widely distributed in angiosperms, also in the Lamiaceae family and particularly in *Salvia* [92]. Chemotaxonomy has always been a field of exploration, so it is not uncommon for phenolic compounds to be studied in this context, investigating their usefulness in characterising and differentiating Taxa or in developing hypotheses of phytochemical evolution [93], in addition to finding better species to obtain natural products. Although it is not an objective of the work, in the case of flavonoids, the comparison exercise is carried out between the three species, to investigate their value as taxonomic markers. They are documented to present different profiles: *S. cinnabarina*, a herb with red flowers and exserted stamens, exhibits seven flavonoids, of which it shares four with *S. lavanduloides*, a herb with blue flowers and inset stamens, and one with *S. longispicata*, a suffrutex with blue flowers and inset stamens. *S. longispicata* has four flavonoids, of which it shares one with *S. cinnabarina* and two with *S. lavanduloides*. The greatest similarity is found between *S. cinnabarina* and *S. lavanduloides*, something that is not expected, given the morphological characteristics of each species and the position they present in the phylogenies [75,93,94] since *S. cinnabarina* is located in a more basal clade with respect to the other two, which are found in more recent and closer clades. We do not consider these results sufficient to evaluate the value of these compounds as taxonomic markers, and we think it is necessary to include a larger number of species in studies with this objective; taking into account that, unlike Old World salvias, studies reporting phenolic compounds for American sages are still scarce.

In order to determine the analgesic potential of *Salvia* species, the methanolic extracts of the three salvias were evaluated using the formalin test. The results show the effect of the extracts at the central and peripheral level, associated with the reduction of nociceptive behaviour in the neurogenic and inflammatory phases.

It is worth noting that the extract of *S. longispicata* reduced nociceptive behavior to a greater extent in both phases. This was possibly due to the chemical differences with respect to the other two salvias. The main difference lies in the presence of a greater number of abietane-type diterpenes. In this regard, the therapeutic effect of tanshinones, carnosic acid and carnosol, isolated from various species of *Salvia*, is reported in various conditions that cause nociceptive pain, associated with inflammation [38,95,96,97].

These results are consistent with the antinociceptive effect observed in other *Salvia* species, such as the ethanolic extract and nor-abietane fruticulin, obtained from *S. lachnostachys*, that had good activity in the formalin test [98]. Additionally, the hydroalcoholic extract and terpenoids isolated from *S. officinalis* produced antinociceptive and anti-inflammatory activity in the Writhing, formalin, and carrageenan tests [38]. Extracts of different polarity, as well as clerodane-type compounds from *S. amarissima* (Syn. *Salvia circinata*), *S. divinorum* Epling & Jativa, *S. semiatrata*, *S. purpurea*, and *S. tiliifolia* promoted significant effects in pain and inflammation tests (Writhing, formalin, hot plate, carrageenan tests, and in a model of fibromyalgia and allodynia) [6,34,35,36,37,99,100].

Several studies have reported the analgesic and anti-inflammatory properties, both in vitro and in vivo models, of flavonoids such as apigenin, kaempferol, quercetin, rutin, and luteolin [101,102,103,104] as well as some phenolic acids such as rosmarinic, sagerinic, syringic, and salvianolic [105].

Among terpenoids, those of the di- type have been little studied regarding their analgesic potential, however, an in vitro study of the anti-inflammatory effect of hardwickiic acid is reported [106]. Finally, regarding triterpenoids, the anti-pain potential of oleanolic and ursolic acids, as well as their derivatives, has been widely studied in nociception models [107,108,109,110,111,112]. All of the above explains why, despite the different chemical profiles of *S. cinnabarina*, *S. lavanduloides*, and *S. longispicata*, all three have antinociceptive effects, since both shared and exclusive metabolites have shown good results in evaluations of their analgesic properties.

The background on the analgesic effect of *Salvia* and the agreement with the results obtained suggest a high potential as a medicinal alternative for pain relief, due to the synergy of terpene and phenolic molecules present in the genus.

Due to the need to know the safety of the plants used in traditional Mexican medicine, an evaluation of the acute toxicity of methanol extracts at a dose of 2000 mg/kg p.o. of *S. cinnabarina*, *S. lavanduloides*, and *S. longispicata* was carried out, the results of which place them at a non-toxic level of use (OECD 2001) [44]. The safety of use of these sages is consistent with the acute toxicity results of other species of the genus, with an LD_50_ > 2000 mg/kg, p.o. calculated for extracts. For example, for the hydroalcoholic extract of *S. officinalis* leaves, an LD_50_ = 44.75 g/kg, p.o. was reported [38]. The infusion of *S. circinata* presented an LD_50_ = 5 g/kg, p.o. [113] and an LD_50_ > 2000 mg/kg, administered intraplantarly (i. p.) [34]. While for *S. hypoleuca* Benth. the LD_50_ = 1800 mg/kg, i. p. [114]. Finally, for the extracts of different polarity from the aerial part of *S. purpurea* and *S. semiatrata*, the LD_50_ was greater than 2000 mg/kg, o.p. [35,36], making them absolutely safe and therefore, good prospects for use as novel analgesics agents.

## 4. Materials and Methods

### 4.1. Drugs and Reagents

Diclofenac (DCF) and 37% formalin were purchased from Merck México (Naucalpan, Mexico, Mexico). Tween 80 and saline solution (SS) were purchased from Sigma-Aldrich (St. Louis, MO, USA). The solvent (methanol HPLC grade) used for extraction was purchased from Tecsiquim, S.A. de C.V. (Mexico City, Mexico). The analysis phytochemicals (methanol, leucine enkephalin, acetonitrile, water, and formic acid) were LC-MS grade and purchased from Sigma-Aldrich.

### 4.2. Collection of Plant Material

One kilogram of the aerial part of each of the sages was collected in the surroundings of Miahuatlán, Oaxaca, in June 2019 (Table 2). Fresh plant material was placed in a drying chamber at 32 °C. Salvias were identified by Ph.D. Martha J. Martínez Gordillo. Voucher specimens of these samples were deposited at the FCME Herbarium of the Faculty of Sciences (FCME), the National Autonomous University of Mexico (UNAM).

### 4.3. Preparations of the Extracts

The plant material of each sage was dried at room temperature and finely ground with a blender. Then, five grams of dried and ground plant material were weighed in quadruplicate in Falcon^®^ conical tubes (Corning Inc., Corning, NY, USA), 50 mL of HPLC grade methanol was added and placed in an ultrasonic bath (Branson Bransonic^®^ Bath 2800, Emerson Electric Co., St. Louis, MO, USA) for 20 min at room temperature and ultrasonic wave frequency of 40 kHz. It was then filtered (medium-pore filter paper) and evaporated to dryness with a rotary evaporator at a temperature of 45 °C (RE100-Pro Digital Rotary Evaporator, DLAB SCIENTIFIC Co., Shunyi, Beijing, China). The dried extracts were stored in amber glass vials away from sunlight and moisture.

### 4.4. Chemical Profiling via Ultra Performance Liquid Chromatography Coupled Mass Spectrometry (UPLC-ESI^+/−^-MS-QTOF)

Chemical profiling of methanol extracts was performed as was previously described by Monribot-Villanueva et al. (2020) [115]. The analysis was carried out using an ultra-high-resolution chromatographic system (ACQUITY UPLC I-Class System, Waters Co., Milford, MA, USA) coupled with a quadrupole time of flight (QTOF) high-resolution mass spectrometer (SYNAPT G2-Si Mass Spectrometry, Waters Co., Milford, MA, USA) with an electrospray ionisation source in positive and negative mode. An ACQUITY UPLC BEH C18 column (Waters Co., Milford, MA, USA) was used, with column and sample temperatures of 40° and 15 °C, respectively. The flow rate was 0.3 mL/min and 5 µL of extract was injected. The mobile phase consisted of a gradient of water and acetonitrile, both with 0.1% formic acid. The gradient conditions were 0–20 min linear gradient 1−99% B, then 20–24 min an isocratic step at 99% B, next 24–25 min a linear gradient 90–1% B, and finally an isocratic step at 1% B for 5 min (total run time 30 min). The mass spectrometer conditions were: Capillary, sampling cone and source offset voltages of 3000, 40, and 80 V, respectively. Source and desolvation temperatures of 120 and 20 °C, respectively. Desolvation gas flow was set at 600 L/h and the nebulizer pressure of 6.5 Bar. The peptide leucine-enkephalin was used as the lock mass (556.2771, [M + H]^+^; 554.2615, [M − H]^−^). The mass acquisition method used was MSe in high-resolution mode (>29,000 *m*/*z* for leucine-enkephalin in both ionisation modes) using a mass range of 50–1200 Da and a scan time of 0.5 s. The collision energies for Function 1 were 6 V and for Function 2 were a ramp from 10 to 30 V. Spectrometric data were acquired and processed with MassLynx version 4.1 and MarkerLynx version 4.1 software (Waters^TM^ Corporation, Milford, MA, USA).

### 4.5. Pharmacological Evaluations

#### 4.5.1. Animals

The pharmacological evaluation was carried out in male CD-1 mice (25–30 g of body weight). The mice were provided by the biotherium of the Faculty of Sciences. Mice were placed in acrylic boxes with water and food ad libitum and kept at a controlled temperature of 22  ±  2  °C, standard humidity (50 ± 5%) and with a 12 h light/dark cycle. All experimental procedures were carried out in accordance with the Official Mexican Standards, NOM-062-ZOO-1999 [116], and International Standards for the Care and Use of Laboratory Animals in Research guidelines. The protocol was accepted by the Committee on Academic Ethics and Scientific Responsibility (CEARC for its acronym in Spanish) of the Faculty of Sciences, UNAM, under the folio PI_2021_08_02_Aguirre. Extracts and the reference drug were suspended in 0.9% SS and Tween 80. All treatments were administered orally (p.o.) in a volume of 10 mL/kg of mouse body weight and were prepared on the day of the experiment.

#### 4.5.2. Acute Toxicity

The toxicity of *Salvia* extracts was evaluated following OECD’s test No. 423 (2001). The experimental groups of three mice were administered a maximum dose of 2000 mg/kg, p.o. with the methanol extracts. The mice were observed for fourteen days to record signs of toxicity such as weight loss, motor incoordination, ataxia, respiratory arrest, or death.

#### 4.5.3. Formalin Test

The experiments were divided into groups of five animals, which received the following treatment: saline solution (SS), reference drug (DFC, 10 mg/kg), and the dose of methanol extract (300 mg/kg, p.o.). Once the acute oral toxicity was evaluated, a wide window of therapeutic activity was obtained in which no toxic effects were observed. This allowed the choice of a single dose of 300 mg/kg, in accordance with the background obtained by the working group, which demonstrates the significant effect in various pain models of extracts of different polarity from various species of *Salvia* (23–25). After 30 min of treatment administration, animals were injected subcutaneously on the intraplantar surface of the right hind limb with 20 µL of 1% formalin to produce a licking behaviour. Individually, the mice were placed inside a glass cylinder, surrounded by mirrors, to facilitate viewing of the behaviour from all angles by the evaluator. Then, the time spent licking the limb administered with the nociceptive agent was measured for 1 min every 5 min for a period of 30 min. Two phases were recorded in this test: neurogenic (0–10 min) and inflammatory (10–30 min) phases. A significant decrease in either phase was interpreted as demonstrative of an antinociceptive effect [117].

### 4.6. Statistical Analysis

Spectrometric data were acquired and processed with the MassLynx v. 4.1 and MarkerLynx v. 4.1 software from Waters (Milford, MA, USA). The intensity of each ion was normalised and filtered relative to the total ion count to generate a data matrix. Such matrix included *m*/*z* values, retention times and normalised peak areas. The mass spectra of the chromatographic peaks were compared with public spectral databases of FooDB, MassBank, LOTUS, Scopus and UNIIQUIM, using a maximum mass error of ±5 ppm as an accuracy criterium of chemical identity. The *m*/*z* dataset underwent pre-processing, which involved centring on the mean and scaling using the Pareto principle. Subsequently, for pattern recognition, a PCA and a heat map were applied using the MetaboAnalyst v. 6.0 (Xia Lab, Montreal, QC, Canada) platform. Data from the antinociceptive activity experiments were statistically analysed using Prism 8 software v. 8.4.3 (GraphPad Software Inc., Boston, MA, USA) and ANOVA, followed by Dunnett’s post hoc test, to compare treatments against the vehicle group. A value of *p* > 0.05 was considered significant.

## 5. Conclusions

The untargeted metabolomic analysis and the review previously carried out on the chemical constituents of salvias allowed for the chemical differentiation of *S. cinnabarina*, *S. lavanduloides*, and *S. longispicata*, of which 46 compounds were identified. In this study, advanced analytical and chemometric techniques were used to identify bioactive compounds and distinctive chemical markers of three Mexican sages, making it one of the few studies that have used the metabolomic technique in Mexico. Likewise, the importance of the synergy of the constituents of terpene and phenolic nature was visualised, which plays an important role in the efficacy and safety of the use of salvias as an alternative therapy in the treatment of pain. The results of this study reinforce the richness of secondary metabolism as well as the therapeutic properties of Mexican *Salvia* species used in Traditional Medicine. It also provides evidence that *S. cinnabarina*, *S. lavanduloides*, and *S. longispicata* may be a source of effective and safe compounds with analgesic potential. Future trials evaluating extracts and isolated compounds from *Salvia* in various biological models are necessary to propose and integrate new drugs into healthcare due to the growing interest in finding alternative therapies.

## Figures and Tables

**Table 1:** Tentative identification of compounds in the methanolic extract of *S. cinnabarina*, *S. lavanduloides* y *S. longispicata*. Retention time (Rt) in minutes. (*m*/*z*) = mass/charge ratio. Mass error in ppm. [M + H]^+^, [M − H]^−^ indicate the identification of compounds in positive and negative mode, respectively. *S. cinnabarina* (*SCi*), *S. lavanduloides* (*SLa*) y *S. longispicata* (*SLo*). n.i. = non-identified. The + symbol indicates the presence of compound.

No.	Rt (min)	(*m*/*z*)	Compound	Adduct	Mass Error (ppm)	*SCi*	*SLa*	*SLo*	Reference
1	0.52	719.1643	Sagerinic acid	[M − H]^−^	4.3	+	n.i	n.i	[45]
2	2.74	563.1403	Schaftoside	[M − H]^−^	0.4	n.i	+	n.i	[46]
3	3.41	197.0449	Syringic acid	[M − H]^−^	−0.5	n.i	n.i	+	[47]
4	3.91	610.1548	Cyanidin 3,5-diglucoside	[M − H]^−^	2.3	n.i	n.i	+	[48]
5	4.21	609.1461	Rutin	[M − H]^−^	0.8	+	+	+	[47]
6	4.29	477.0678	Miquelianin	[M − H]^−^	1.9	n.i	n.i	+	[46]
7	4.33	463.0881	Quercetin-O-hexoside	[M − H]^−^	0.8	n.i	+	+	[5]
8	4.46	461.0725	Luteolin-7-O-glucuronide	[M − H]^−^	1.1	+	n.i	n.i	[45]
9	4.67	521.1296	Salviaflaside	[M − H]^−^	0.2	+	n.i	+	[45]
10	4.72	477.1045	Rhamnetin 3-glucoside	[M − H]^−^	2.5	+	+	n.i	[46]
11	4.79	301.0344	Quercetin	[M − H]^−^	−1.3	n.i	+	n.i	[34]
12	5.08	269.0448	Apigenin	[M − H]^−^	−0.7	+	n.i	n.i	[49]
13	5.20	359.0775	Rosmarinic acid	[M − H]^−^	2.2	+	+	+	[45]
14	5.37	493.1141	Salvianolic acid A	[M − H]^−^	1.2	n.i	+	n.i	[45]
15	5.39	285.0397	Luteolin	[M − H]^−^	−0.7	+	+	n.i	[45]
16	5.60	315.0510	Pedalitin	[M − H]^−^	1.6	n.i	+	+	[50]
17	5.92	343.0816	5,6-Dihydroxy-7,3’,4’-trimethoxyflavone	[M − H]^−^	−0.6	+	n.i	n.i	[49]
18	6.22	287.0554	Kaempferol	[M + H]^+^	−0.7	+	n.i	n.i	[51]
	6.22	285.0398	Kaempferol	[M − H]^−^	−0.3	n.i	+	n.i.	[51]
19	6.54	593.1865	Amarisolide F	[M − H]^−^	−0.8	n.i	+	+	[49]
20	6.74	355.1182	Dugesin G	[M − H]^−^	0.0	n.i	+	n.i.	[52]
21	6.75	337.1092	Salvixalapadiene	[M − H]^−^	4.7	n.i	+	+	[53]
22	6.81	349.2008	2α,6α-Dihydroxy-patagonol	[M − H]^−^	−2.0	n.i	+	n.i	[54]
23	6.90	347.1863	15,16-Diol-15,16-dihydro-hardwickiic acid	[M − H]^−^	1.4	+	n.i	n.i	[55]
24	7.03	357.1333	Splenolide A	[M − H]^−^	−1.4	n.i	+	n.i	[56]
25	7.20	371.1132	Salvianduline B	[M − H]^−^	0.3	n.i	+	n.i	[57]
26	8.14	399.1435	Salvimexicanolide	[M − H]^−^	−2.2	n.i	+	n.i	[58]
27	8.17	347.1864	16-Hydroxycarnosic acid	[M − H]^−^	1.7	n.i	+	+	[59]
28	8.20	345.1711	16-Hydroxycarnosol	[M − H]^−^	2.6	n.i		+	[60]
29	8.30	333.2066	11,12,16,20-Tetrahydroxy-abieta-8,11,13-triene	[M − H]^−^	0.0	+	n.i	n.i	[59]
30	9.23	315.1954	20-Deoxocarnosol	[M − H]^−^	−1.9	+	n.i	n.i	[59]
31	9.91	389.1961	16-Acetoxycarnosic acid	[M − H]^−^	−0.8		+	n.i	[59]
32	9.94	329.1752	Carnosol	[M − H]^−^	−0.3	+	n.i	n.i	[5]
33	10.10	331.1909	Carnosic acid	[M − H]^−^	0.0	+	n.i	n.i	[59]
34	10.39	331.1914	5,6-Dihydro-6α-hydroxysalviasperanol	[M − H]^−^	1.5	+	n.i	+	[61]
35	10.58	329.1757	19-Deoxyicetexone	[M]^−^	1.2	n.i	n.i	+	[62]
36	10.66	317.2120	Demethylsalvicanol	[M − H]^−^	0.9	+	n.i	n.i	[61]
37	11.33	389.1971	7α-Acetoxy-6,7-dihydroicetexone	[M]^−^	1.8	n.i	+	n.i	[41]
38	11.42	301.1809	Royleanone	[M − H]^−^	1.6	n.i	n.i	+	[63]
39	11.43	343.1549	Rosmadial	[M − H]^−^	1.2	n.i	n.i	+	[60]
40	11.47	355.1542	Tilifolidione	[M − H]^−^	−1.1	n.i	n.i	+	[63]
41	11.63	301.2170	3,4-Secoisopimar-4(18),7,15-trien-3-oic acid	[M − H]^−^	0.7	+	n.i	n.i	[64]
42	12.16	317.2110	6β-Hydroxy-*trans*-communic acid	[M − H]^−^	−2.2	+	n.i	n.i	[65]
43	12.21	455.3161	Dihydroxy-24-nor-4(23),12-oleanadien-28-oic acid	[M − H]^−^	0.0	+	n.i	n.i	[66]
44	12.34	275.2006	3-Hydroxyestran-17-one	[M − H]^−^	−1.8	+	+	+	[67]
45	13.29	469.3325	11β-Hydroxy-3-oxo-urs-12-en-28-oic acid	[M − H]^−^	1.5	+	+	+	[68]
46	13.44	471.3481	2-Hydroxyursolic acid	[M − H]^−^	1.5	+	n.i	+	[69]

**Table 2:** Altitude, geographical position and voucher number of *Salvia* species.

Species	Elevation (masl)	Geographical Position	Voucher Number FCME
*S. cinnabarina*	2712	16°06′42.6″ N, 96°28′22.1″ W	184,736
*S. lavanduloides*	2712	16°06′42.6″ N, 96°28′22.1″ W	184,738
*S. longispicata*	2301	19°14′31.2″ N, 94°38′25.8″ W	184,737

## Data Availability

Data are contained within the article.

## References

[1] Wu Y.B., Ni Z.Y., Shi Q.W., Dong M., Kiyota H., Gu Y.C., Cong B. (2012). Constituents from *Salvia* species and their biological activities. Chem. Rev..

[2] Irtegun K.S., Fidan H.S., Yener I., Mete N., Ertas A., Topcu G., Kolak U. (2022). Investigation of cytotoxic and apoptotic effects of 63 compounds obtained from *Salvia* species: Promising anticancer agents. J. Food Biochem..

[3] Cuevas-Morales C., Ortiz-Mendoza N., Martínez-Gordillo M.J., Basurto-Peña F.A., Palma-Tenango M., Aguirre-Hernández E. (2024). Mexico’s sage richness, traditional uses and chemical composition: A review. Agro Product..

[4] Zhumaliyeva G., Zhussupova A., Zhusupova G.E., Błońska-Sikora E., Cerreto A., Omirbekova N., Zhunusbayeva Z., Gemejiyeva N., Ramazanova M., Wrzosek M. (2023). Natural compounds of *Salvia* L. genus and molecular mechanism of their biological activity. Biomedicines.

[5] Afonso A.F., Pereira O.R., Fernandes Â.S.F., Calhelha R.C., Silva A.M.S., Ferreira I.C.F.R., Cardoso S.M. (2019). The health-benefits and phytochemical profile of *Salvia apiana* and *Salvia farinacea* var. victoria blue decoctions. Antioxidants.

[6] Moreno-Pérez F., Hernández-León A., Valle-Dorado M.G., Cano-Martínez A., Narváez-González F., Aguirre-Hernández E., Salgado-Ceballos H., González-Trujano M.E. (2021). Neo-clerodane diterpenic influence in the antinociceptive and anti-inflammatory properties of *Salvia circinnata* Cav. J. Ethnopharmacol..

[7] Feng J.H., Jung J.S., Hwang S.H., Lee S.K., Lee S.Y., Kwak Y.G., Kim D.H., Song C.Y., Kim M.J., Suh H.W. (2022). The Mixture of *Agrimonia Pilosa* Ledeb. and *Salvia Miltiorrhiza* Bunge. extract produces analgesic and anti-inflammatory effects in a collagen-induced arthritis mouse model. Anim. Cells Syst..

[8] Gkioni M.D., Zeliou K., Dimaki V.D., Trigas P., Lamari F.N. (2022). GC-MS and LC-DAD-MS phytochemical profiling for characterization of three native *Salvia* taxa from Eastern Mediterranean with antiglycation properties. Molecules.

[9] Jung H.J., Song Y.S., Lim C.J., Park E.H. (2009). Anti-inflammatory, anti-angiogenic and anti-nociceptive activities of an ethanol extract of *Salvia plebeia* r. brown. J. Ethnopharmacol..

[10] Rodrigues M.R.A., Kanazawa L.K.S., Neves T.L.M.D., Silva C.F.D., Horst H., Pizzolatti M.G., Werner M.F.D.P. (2012). Antinociceptive and anti-inflammatory potential of extract and isolated compounds from the leaves of *Salvia officinalis* in mice. J. Ethnopharmacol..

[11] Balsalobre N.d.M., dos Santos-Procopio E., Oliveira C.S., Neves S.C., Verdan M.H., Silva-Filho S.E., Oliveira R.J., Stefanello M.É.A., Kassuya C.A.L. (2024). The anti-arthritic potential of the ethanolic extract of *Salvia lachnostachys* benth. leaves and icetexane dinor-diterpenoid fruticuline b. Pharmaceuticals.

[12] Martínez-Gordillo M., Bedolla-García B., Cornejo-Tenorio G., Fragoso-Martínez I., García-Peña M.D.R., González-Gallegos J.G., Lara-Cabrera S.I., Zamudio S. (2017). Lamiaceae de México. Bot. Sci..

[13] Cornejo-Tenorio G., Ibarra-Manríquez G. (2011). México diversity and distribution of the genus *Salvia* (Lamiaceae) in Michoacan, Mexico. Rev. Mex. Biodivers..

[14] González-Gallegos J.G., Bedolla-García B.Y., Cornejo-Tenorio G., Fernández-Alonso J.L., Fragoso-Martínez I., García-Peña M.D.R., Harley R.M., Klitgaard B., Martínez-Gordillo M.J., Wood J.R.I. (2020). Richness and distribution of *Salvia* Subg. Calosphace (Lamiaceae). Int. J. Plant. Sci..

[15] Martínez M. (1975). Flora Medicinal del Estado de México.

[16] Argueta A., Cano L., Rodarte M., Gallardo C. (1994). Atlas de las Plantas de la Medicina Tradicional Mexicana.

[17] Cahill J.P. (2003). Ethnobotany of chia, *Salvia hispanica* L. (Lamiaceae). Econ. Bot..

[18] Cornejo G., Ibarra G. (2008). Flora Ilustrada de la Reserva de la Mariposa Monarca.

[19] Ortiz-Mendoza N., Aguirre-Hernández E., Fragoso-Martínez I., González-Trujano M.E., Basurto-Peña F.A., Martínez-Gordillo M.J. (2022). A review on the ethnopharmacology and phytochemistry of the neotropical sages (*Salvia* Subgenus *Calosphace*; Lamiaceae) emphasizing mexican species. Front. Pharmacol..

[20] Jenks A., Kim S.C. (2013). Medicinal plant complexes of *Salvia* subgenus *Calosphace* an ethnobotanical study of new world sages. J. Ethnopharmacol..

[21] Mendoza B. (1983). Estudio Etnobotánico de Plantas Medicinales en el Ejido Santa Ana, Teoloyucan, Estado de México. Bachelor’s Thesis.

[22] Estrada E. (1984). Las Plantas Medicinales y los Sistemas Tradicionales de Curación del Municipio de Dr. Mora, Guanajuato. Bachelor’s Thesis.

[23] Jiménez J. (1994). Plantas Medicinales de San Juan Tepecoculco, Municipio de Atlautla de Victoria, Estado de México. Bachelor’s Thesis.

[24] Martínez M.A., Evangelista V., Mendoza M., Morales G., Toledo G., Wong A. (1995). Catálogo de Plantas Útiles de la Sierra Norte de Puebla, México.

[25] Lozano G. (1996). Plantas medicinales utilizadas por los mazahuas del municipio de San Felipe del Progreso, estado de México. Bachelor’s Thesis.

[26] González M., López L., González S., Tena J. (2004). Plantas Medicinales del Estado de Durango y Zonas Aledañas.

[27] Bello M., Salgado R. (2007). Plantas medicinales de la comunidad indígena Nuevo San Juan Parangaricutiro, Michoacán, México. Biológicas.

[28] Andrade A. (2009). Ethnobotanical study of the medicinal plants from Tlanchinol, Hidalgo, México. J. Ethnopharmacol..

[29] Molina J., Galván R., Patiño A., Fernández R. (2012). Plantas medicinales y listado florístico preliminar del municipio de Huasca de Ocampo, Hidalgo, México. Polibotánica.

[30] Cruz-Pérez A., Barrera J., Bernal L., Bravo D., Rendón B. (2021). Actualized inventory of medicinal plants used in traditional medicine in Oaxaca, Mexico. J. Ethnobiol. Ethnomed..

[31] Bozzini M.F., Pieracci Y., Ascrizzi R., Najar B., D’Antraccoli M., Ciampi L., Peruzzi L., Turchi B., Pedonese F., Alleva A. (2023). Chemical composition and antimicrobial activity against the Listeria monocytogenes of essential oils from seven *Salvia* species. Foods.

[32] Lu Y., Foo L.Y. (2002). Polyphenolics of *Salvia*—A review. Phytochemistry.

[33] Campos-Xolalpa N., Pérez-Gutiérrez S., Pérez-González C., Mendoza-Pérez J., Alonso-Castro A., Akhtar M.S., Swamy M.K. (2018). Terpenes of the genus Salvia: Cytotoxicity and antitumoral effects. Anticancer Plants: Natural Products and Biotechnological Implements.

[34] Moreno-Pérez G.F., González-Trujano M.E., Martínez-Gordillo M.J., Miguel-Chávez R.S., Basurto-Peña F.A., Dorazco-González A., Aguirre-Hernández E. (2019). Amarisolide A and pedalitin as bioactive compounds in the antinociceptive effects of *Salvia circinata* (Lamiaceae). Bot. Sci..

[35] Cuevas-Morales C., Zavala-Ocampo L.M., Miguel-Chávez R.S., González-Trujano M.E., Basurto-Peña F.A., Muñoz-Ocotero V., Aguirre-Hernández E. (2022). Pharmacological evaluation of the antinociceptive activity and phytochemical analysis of active extracts of *Salvia purpurea* Cav. Bot. Sci..

[36] Ortiz-Mendoza N., Zavala-Ocampo L.M., Martínez-Gordillo M.J., González-Trujano M.E., Peña F.A.B., Bazany-Rodríguez I.J., Chávez J.A.R., Dorazco-González A., Aguirre-Hernández E. (2020). Antinociceptive and anxiolytic-like effects of a neo-clerodane diterpene from *Salvia semiatrata* Aerial Parts. Pharm. Biol..

[37] González-Chávez M.M., Alonso-Castro A.J., Zapata-Morales J.R., Arana-Argáez V., Torres-Romero J.C., Medina-Rivera Y.E., Sánchez-Mendoza E., Pérez-Gutiérrez S. (2018). Anti-Inflammatory and antinociceptive effects of tilifodiolide, isolated from *Salvia tiliifolia* Vahl (Lamiaceae). Drug Dev. Res..

[38] Sousa A.K., Brito M.V., Prudêncio R.D.S., Sousa S.G., Carvalho A.D.S., Silva T.M.L.D., Almeida V.P.A., Sousa J.J.D.S., Gomes P.R.C., Marques R.A. (2024). The annonalide diterpene extracted from *Casimirella ampla* (Miers) reduces inflammatory and antinociceptive events in general models of inflammation. J. Ethnopharmacol..

[39] Hirota B.C.K., Paula C.D.S., De Oliveira V.B., Da Cunha J.M., Schreiber A.K., Ocampos F.M., Miguel M.D. (2016). Phytochemical and antinociceptive, anti-inflammatory, and antioxidant studies of *Smilax larvata* (Smilacaceae). Evid. Based Complement. Altern. Med..

[40] do Nascimento J.E.T., de Morais S.M., de Lisboa D.S., de Oliveira Sousa M., Santos S.A.A.R., Magalhães F.E.A., Campos A.R. (2018). The orofacial antinociceptive effect of kaempferol-3-O-rutinoside, isolated from the plant *Ouratea fieldingiana*, on adult zebrafish (Danio rerio). Biomed. Pharmacother..

[41] Esquivel B. (2008). Rearranged clerodane and abietane derived diterpenoids from American *Salvia* species. Nat. Prod. Commun..

[42] Ortiz-Mendoza N., San Miguel-Chávez R., Martínez-Gordillo M.J., Basurto-Peña F.A., Palma-Tenango M., Aguirre-Hernández E. (2023). Variation in terpenoid and flavonoid content in different samples of *Salvia Semiatrata* collected from Oaxaca, Mexico, and its effects on antinociceptive activity. Metabolites.

[43] Royal Botanic Gardens Plants of the World Online POWO. https://powo.science.kew.org/.

[44] OECD Gideline for Testing of Chemicals Acute Oral Toxicity—Acute Toxic Class Method 2001. http://www.oecd.org/chemicalsafety/risk-assessment/1948378.pdf.

[45] Pereira O.R., Catarino M.D., Afonso A.F., Silva A.M.S., Cardoso S.M. (2018). *Salvia elegans*, *Salvia greggii* and *Salvia officinalis* Decoctions: Antioxidant activities and inhibition of carbohydrate and lipid metabolic enzymes. Molecules.

[46] Bisio A., Romussi G., Ciarallo G., De Tommasi N. (1997). Flavonoide und triterpenoide aus *Salvia blepharophylla* Brandegee ex. Epling. Pharmazie.

[47] Hamad G.M., Mohdaly A.A.A., El-Nogoumy B.A., Ramadan M.F., Hassan S.A., Zeitoun A.M. (2021). Detoxification of aflatoxin b1 and ochratoxin a using *Salvia farinacea* and *Azadirachta indica* water extract and application in meat products. Appl. Biochem. Biotechnol..

[48] Saito N., Harborne J.B. (1992). Correlations between anthocyanin type, pollinator and flower colour in the labiatae. Phytochemistry.

[49] Calzada F., Bautista E., Barbosa E., Salazar-Olivo L.A., Alvidrez-Armendáriz E., Yepez-Mulia L. (2020). Antiprotozoal activity of secondary metabolites from *Salvia circinata*. Rev. Bras. Farmacogn..

[50] Bisio A., Corallo A., Gastaldo P., Romussi G., Ciarallo G., Fontana N., De Tommasi N., Mo F.U. (1999). Glandular hairs and secreted material in *Salvia blepharophylla* Brandegee Ex Epling grown in Italy. Ann. Bot..

[51] Kamel M.S., Desoky E.K., Abdallah O.M., Bishay D.W. (1992). Flavonol glycosides from leaves of *Salvia farinacea* Benth. B-FOPCU.

[52] Gang X., Fang Z., Xian-Wen Y., Juan Z., Li-Xin Y., Shen X.L., Hu Y.J., Zhao Q.S. (2011). Neo-clerodane diterpenoids from *Salvia dugesii* and their bioactive studies. Nat. Prod. Bioprospect..

[53] Esquivel B., Tello R., Sánchez A.A. (2005). Unsaturated diterpenoids with a novel carbocyclic skeleton from *Salvia xalapensis*. J. Nat. Prod..

[54] Torres M.J.P. (2021). Aislamiento y Elucidación Estructual de los Metabolitos Secundarios de *Salvia gesneriflora* y *S. guevarae*. Implicaciones Quimiotaxonómicas. Master’s Thesis.

[55] Bisio A., Schito A.M., Ebrahimi S.N., Hamburger M., Mele G., Piatti G., Romussi G., Dal Piaz F., De Tommasi N. (2015). Antibacterial compounds from *Salvia adenophora* Fernald (Lamiaceae). Phytochemistry.

[56] Bautista E., Toscano A., Calzada F., Díaz E., Yépez-Mulia L., Ortega A. (2013). Hydroxyclerodanes from *Salvia shannoni*. J. Nat. Prod..

[57] Ortega A., Cardenas J., Toscano A., Maldonado E., Aumelas A., Rose M., Jankowskq C. (1991). Salviandulines A and B. Two secoclerodane diterpenoids from *Salvia lavanduloides*. Phytochemistry.

[58] Frontana-Uribe B.A., Escárcega-Bobadilla M.V., Estrada-Reyes R., Morales-Serna J.A., Salmón M., Cárdenas J. (2011). A New languidulane diterpenoid from *Salvia mexicana* var. mexicana. Molecules.

[59] Luis J.G., Andrés L.S. (1993). C-16 Hydroxylated abietane diterpenes from *Salvia mellifera*. Phytochemistry.

[60] Guerrero I.C., Andrés L.S., León L.G., Machín R.P., Padrón J.M., Luis J.G., Delgadillo J. (2006). Abietane diterpenoids from *Salvia pachyphylla* and *S. clevelandii* with cytotoxic activity against human cancer cell lines. J. Nat. Prod..

[61] Esquivel B., Flores M., Hernandez-Ortega S., Toscano R.A., Ramamoorthy T.P. (1995). Abietane and icetexane diterpenoids from the roots of *Salvia aspera*. Phytochemistry.

[62] Esquivel B., Calderon J.S., Flores E., Rosas Rivera R. (1997). Abietane and icetexane diterpenoids from *Salvia ballotaeflora* and *Salvia axillaris*. Phytochemistry.

[63] Esquivel B., Sanchez A.A. (2005). Rearranged icetexane diterpenoids from the roots of *Salvia thymoides* (Labiatae). Nat. Prod. Res..

[64] Romussi G., Ciarrallo G., Bisio A., Fontana N., De Simone F., De Tommasi N., Mascolo N., Pinto L. (2001). A new diterpeoid with astispasmodic activity from *Salvia cinnabarina*. Planta Med..

[65] Bustos-Brito C., Nieto-Camacho A., Hernández-Ortega S., Rivera-Chávez J., Quijano L., Esquivel B. (2020). Structural elucidation of malonylcommunol and 6β-hydroxy-trans-communic acid, two undescribed diterpenes from *Salvia cinnabarina*. First examples of labdane diterpenoids from a mexican *Salvia* species. Molecules.

[66] Ballesta-Acosta M.C., Pascual-Villalobos M.J., Rodríguez B. (2002). A New 24-nor-oleanane triterpenoid from *Salvia carduacea*. J. Nat. Prod..

[67] Serrano-Vega R., Pérez-González C., Alonso-Castro Á., Zapata-Morales J., Pérez-Gutiérrez S. (2020). Anti-inflammatory and antinociceptive activities of *Salvia keerlii*. Pharmacogn. Mag..

[68] Luis J.G., Andrés L.S. (1999). New ursane type triterpenes from *Salvia mellifera* Greene. Nat. Prod. Lett..

[69] Pereda-Miranda R., Hernández L., Lopez R. (1992). A novel antimicrobial abietane-type diterpene from *Salvia albocaerulea*. Lett. Planta Med..

[70] Wei Y.K., Wang Q., Huang Y.B. (2015). Species diversity and distribution of *Salvia* (Lamiaceae). Biodivers. Sci..

[71] Doğan M., Akıcı N., Diken M.E., Doğan S., Yilmaz-Kardas B., Dirmenci T. (2021). Biological activities of some *Salvia* species. Z. Naturforsch. C J. Biosci..

[72] Shojaeifard Z., Hemmateenejad B., Jassbi A.R. (2021). Chemometrics-based LC-UV-ESIMS analyses of 50 *Salvia* species for detecting their antioxidant constituents. J. Pharm. Biomed. Anal..

[73] Rose J.P., Kriebel R., Kahan L., DiNicola A., González-Gallegos J.G., Celep F., Lemmon E.M., Lemmon A.R., Sytsma K.J., Drew B.T. (2021). Sage insights into the phylogeny of *Salvia*: Dealing with sources of discordance within and across genomes. Front. Plant. Sci..

[74] Epling C. (1939). A revision of *Salvia* subgenus *Calosphace*. Repertorium Specierum Novarum Regni Vegetalis.

[75] Lara-Cabrera S.I., Perez-Garcia M.d.l.L., Maya-Lastra C.A., Montero-Castro J.C., Godden G.T., Cibrian-Jaramillo A., Fisher A.E., Porter J.M. (2021). Phylogenomics of *Salvia* L. subgenus *Calosphace* (Lamiaceae). Front. Plant Sci..

[76] Al-Jaber H.I., Shakya A.K., Elagbar Z.A. (2020). HPLC profiling of selected phenolic acids and flavonoids in *Salvia eigii*, *Salvia hierosolymitana* and *Salvia viridis* growing wild in Jordan and their in vitro antioxidant activity. PeerJ.

[77] Salinas-Arellano E., Pérez-Vásquez A., Rivero-Cruz I., Torres-Colin R., González-Andrade M., Rangel-Grimaldo M., Mata R. (2020). Flavonoids and terpenoids with PTP-1B inhibitory properties from the infusion of *Salvia amarissima* Ortega. Molecules.

[78] Rashwan H.M., Mohammed H.E., El-Nekeety A.A., Hamza Z.K., Abdel-Aziem S.H., Hassan N.S., Abdel-Wahhab M.A. (2021). Bioactive phytochemicals from *Salvia officinalis* attenuate cadmium-induced oxidative damage and genotoxicity in rats. Environ. Sci. Pollut. Res. Int..

[79] Hitl M., Kladar N., Gavarić N., Božin B. (2021). Rosmarinic acid-human pharmacokinetics and health benefits. Planta Med..

[80] Kernou O.N., Azzouz Z., Madani K., Rijo P. (2023). Application of rosmarinic acid with its derivatives in the treatment of microbial pathogens. Molecules.

[81] Liu H., Xu Q., Wufuer H., Li Z., Sun R., Jiang Z., Dou X., Fu Q., Campisi J., Sun Y. (2024). Rutin is a potent senomorphic agent to target senescent cells and can improve chemotherapeutic efficacy. Aging Cell.

[82] Bais H.P., Walker T.S., Schweizer H.P., Vivanco J.M. (2002). Root specific elicitation and antimicrobial activity of rosmarinic acid in hairy root cultures of sweet basil (*Ocimum basilicum* L.). Plant Physiol. Biochem..

[83] Petersen M., Simmonds M.S.J. (2003). Rosmarinic acid. Phytochemistry.

[84] Hafeez M.B., Zahra N., Zahra K., Raza A., Khan A., Shaukat K., Khan S. (2021). Brassinosteroids: Molecular and physiological responses in plant growth and abiotic stresses. Plant Stress.

[85] Bajguz A., Hayat S. (2009). Effects of brassinosteroids on the plant responses to environmental stresses. Plant Physiol. Biochem..

[86] Bartwal A., Mall R., Lohani P., Guru S.K., Arora S. (2013). Role of secondary metabolites and brassinosteroids in plant defense against environmental stresses. J. Plant Growth Regul..

[87] Koutsoulas A., Čarnecká M., Slanina J., Tóth J., Slaninová I. (2019). Characterization of phenolic compounds and antiproliferative effects of *Salvia pomifera* and *Salvia fruticosa* Extracts. Molecules.

[88] Shyamal K.J., Gorai D., Roy R. (2016). *Salvia* genus and triterpenoids. Int. J. Pharm. Sci. Res..

[89] Krol A., Kokotkiewicz A., Luczkiewicz M. (2022). White sage (*Salvia apiana*)—A ritual and medicinal plant of the Chaparral: Plant characteristics in comparison with other *Salvia* species. Planta Med..

[90] González-Cortazar M., Salinas-Sánchez D.O., Herrera-Ruiz M., Román-Ramos D.C., Zamilpa A., Jiménez-Ferrer E., Ble-González E.A., Álvarez-Fitz P., Castrejón-Salgado R., Pérez-García M.D. (2022). Eupatorin and salviandulin-A, with antimicrobial and anti-inflammatory effects from *Salvia lavanduloides* Kunth leaves. Plants.

[91] Bisio A., Pagano B., Romussi A., Bruno O., De Tommasi N., Romussi G., Mattia C.A. (2007). Relative stereochemistry of a diterpene from *Salvia cinnabarina*. Molecules.

[92] Moshari-Nasirkandi A., Iaccarino N., Romano F., Graziani G., Alirezalu A., Alipour H., Amato J. (2024). Chemometrics-based analysis of the phytochemical profile and antioxidant activity of *Salvia* species from Iran. Sci. Rep..

[93] Frezza C., Venditti A., Giuliani C., Foddai S., Cianfaglione K., Maggi F., Fico G., Guiso M., Nicoletti M., Bianco A. (2021). Occurrence of flavonoids in different Lamiaceae taxa for a preliminary study on their evolution based on phytochemistry. Biochem. Syst. Ecol..

[94] Fragoso-Martínez I., Martínez-Gordillo M., Salazar G.A., Sazatornil F., Jenks A.A., García Peña M.d.R., Barrera-Aveleida G., Benitez-Vieyra S., Magallón S., Cornejo-Tenorio G. (2018). Phylogeny of the neotropical sages (*Salvia* subg. Calosphace; Lamiaceae) and insights into pollinator and area shifts. Plant Syst. Evol..

[95] Maione F., Cantone V., Pace S., Chini M.G., Bisio A., Romussi G., Pieretti S., Werz O., Koeberle A., Mascolo N. (2017). Anti-inflamatory ans analgesic activity of carnosol and carnosic acid in vivo and in vitro and in silico analysis of their target interactions. Br. J. Pharmacol..

[96] Jiang Z., Gao W., Huang L. (2019). Tanshinones, critical pharmacological components in *Salvia Miltiorrhiza*. Front. Pharmacol..

[97] Di Cesare Mannelli L., Piccolo M., Maione F., Ferraro M.G., Irace C., De Feo V., Ghelardini C., Mascolo N. (2018). tanshinones from *Salvia miltiorrhiza* bunge revert chemotherapy-induced neuropathic pain and reduce glioblastoma cells malignancy. Biomed. Pharmacother..

[98] Santos J.A., Piccinelli A.C., Formagio M.D., Oliveira C.S., Santos E.P.D., Stefanello M.É.A., Junior U.L., Oliveira R.J., Sugizaki M.M., Kassuya C.A.L. (2017). Antidepressive and antinociceptive effects of ethanolic extract and fruticuline a from *Salvia lachnostachys* benth leaves on rodents. PLoS ONE.

[99] Tlacomulco-Flores L.L., Déciga-Campos M., González-Trujano M.E., Carballo-Villalobos A.I., Pellicer F. (2020). Antinociceptive effects of *Salvia divinorum* and bioactive salvinorins in experimental pain models in mice. J. Ethnopharmacol..

[100] Moreno-Pérez G.F., González-Trujano M.E., Hernández-León A., Valle-Dorado M.G., Valdés-Cruz A., Alvarado-Vásquez N., Aguirre-Hernández E., Salgado-Ceballos H., Pellicer F. (2023). antihyperalgesic and antiallodynic effects of amarisolide a and *Salvia amarissima* Ortega in experimental fibromyalgia-type pain. Metabolites.

[101] Büyükokuroglu M., Berashvili M.E., Altinkeser A. (2008). Antiinflammatory and antinociceptive properties of luteolin diglucuronide and apigenin diglucuronide obtained from *Perilla nankinensis*. Asian J. Chem..

[102] De Melo G.O., Malvar D.d.C., Vanderlinde F.A., Rocha F.F., Pires P.A., Costa E.A., de Matos L.G., Kaiser C.R., Costa S.S. (2009). Antinociceptive and anti-inflammatory kaempferol glycosides from *Sedum dendroideum*. J. Ethnopharmacol..

[103] Martínez A.L., González-Trujano M.E., Aguirre-Hernández E., Moreno J., Soto-Hernández M., López-Muñoz F.J. (2009). Antinociceptive activity of *Tilia americana* var. mexicana inflorescences and quercetin in the formalin test and in an arthritic pain model in rats. Neuropharmacology.

[104] El Shoubaky G.A., Abdel-Daim M.M., Mansour M.H., Salem E.A. (2016). Isolation and identification of a flavone apigenin from marine red alga *Acanthophora spicifera* with antinociceptive and anti-inflammatory activities. J. Exp. Neurosci..

[105] Boonyarikpunchai W., Sukrong S., Towiwat P. (2014). Antinociceptive and anti-inflammatory effects of rosmarinic acid isolated from *Thunbergia Laurifolia* Lindl. Pharmacol. Biochem. Behav..

[106] Símaro G.V., Lemos M., Mangabeira da Silva J.J., Ribeiro V.P., Arruda C., Schneider A.H., Wagner de Souza Wanderley C., Carneiro L.J., Mariano R.L., Ambrósio S.R. (2021). Antinociceptive and anti-inflammatory activities of *Copaifera pubiflora* benth oleoresin and its major metabolite ent-hardwickiic acid. J. Ethnopharmacol..

[107] González-Trujano M.E., Ventura-Martínez R., Chávez M., Díaz-Reval I., Pellicer F. (2012). Spasmolytic and antinociceptive activities of ursolic acid and acacetin identified in *Agastache mexicana*. Planta Med..

[108] Park S.H., Sim Y.B., Kang Y.J., Kim S.S., Kim C.H., Kim S.J., Suh H.W. (2013). Mechanisms involved in the antinociceptive effects of orally administered oleanolic acid in the mouse. Arch. Pharm. Res..

[109] Verano J., González-Trujano M.E., Déciga-Campos M., Ventura-Martínez R., Pellicer F. (2013). Ursolic acid from *Agastache mexicana* aerial parts produces antinociceptive activity involving TRPV1 receptors, CGMP and a serotonergic synergism. Pharmacol. Biochem. Behav..

[110] Déciga-Campos M., Cortés A., Pellicer F., Díaz-Reval I., González-Trujano M.E. (2014). Isobolographic analysis of the antinociceptive interaction between ursolic acid and diclofenac or tramadol in mice. Planta Med..

[111] Bednarczyk-Cwynar B., Wachowiak N., Szulc M., Kaminska E., Bogacz A., Bartkowiak-Wieczorek J., Zaprutko L., Mikolajczak P.L. (2016). Strong and long-lasting antinociceptive and anti-inflammatory conjugate of naturally occurring oleanolic acid and aspirin. Front. Pharmacol..

[112] Rali S., Oyedeji O.O., Aremu O.O., Oyedeji A.O., Nkeh-Chungag B.N. (2016). Semisynthesis of derivatives of oleanolic acid from *Syzygium aromaticum* and their antinociceptive and anti-inflammatory properties. Mediat. Inflamm..

[113] Flores-Bocanegra L., González-Andrade M., Bye R., Linares E., Mata R. (2017). α-Glucosidase inhibitors from *Salvia circinata*. J. Nat. Prod..

[114] Karami M., Shamerani M.A., Alemy S.H., Gohari A.R., Ehsani V.S. (2013). Comparison antinociceptive activity of the aqueous methanolic extracts of *Salvia hypoleuca* and *Phytolacca americana* in mice. Eur. Rev. Med. Pharmacol. Sci..

[115] Monribot-Villanueva J.L., Rodríguez-Fuentes J.S., Landa-Cansigno C., Infante-Rodríguez D.A., Díaz-Abad J.P., Guerrero-Analco J.A. (2020). Comprehensive profiling and identification of bioactive components from methanolic leaves extract of *Juniperus deppeana* and its in vitro antidiabetic activity. Can. J. Chem..

[116] Norma Oficial Mexicana NOM-062-ZOO-1999. https://www.gob.mx.

[117] Tjølsen A., Berge O.G., Hunskaar S., Rosland J.H., Hole K. (1992). The Formalin Test: An Evaluation of the Method. Pain.

